# Molecular Cargo of Exosomes in Prostate Cancer: A Multi-Omics Perspective on Liquid Biopsies

**DOI:** 10.3390/genes16121437

**Published:** 2025-12-01

**Authors:** Roxana Andra Coman, Andreea Nutu, Stefan Strilciuc, Liviuta Budisan, Ioana Berindan-Neagoe

**Affiliations:** 1 Department of Urology, MedLife Humanitas Hospital, 400664 Cluj-Napoca, Romania; dr.roxanacoman@yahoo.com; 2 Genomics Department, MEDFUTURE Institute of Biomedical Research, Iuliu-Hatieganu University of Medicine and Pharmacy, 400347 Cluj-Napoca, Romania; 3 Doctoral School, Iuliu-Hatieganu University of Medicine and Pharmacy, 400347 Cluj-Napoca, Romania; 4 Academy of Medical Sciences, 030167 Bucharest, Romania

**Keywords:** prostate cancer, exosomes, liquid biopsy, miRNAs, genomic cargo

## Abstract

Prostate cancer is one of the most common cancers affecting men, and finding better ways to detect and monitor it remains a top priority in oncology. In recent years, scientists have focused their attention on different classes of extracellular bodies, among them the small ones called exosomes. Exosomes are nanoscale extracellular vesicles (30–200 nm) released into body fluids, where they transport molecular cargo reflective of their cell of origin. Instead of serving as liquid biopsies themselves, exosomes present in accessible fluids such as plasma and urine can be analyzed as part of minimally invasive liquid biopsy strategies without the need for surgery or tissue sampling. In prostate cancer, exosomes are not just passive carriers: they actively influence how cancer grows, spreads, and responds to treatment. Exosomes can be extracted from simple fluid samples, opening the door to faster, safer, and more personalised approaches to diagnosis and care. Exosome content is analysed for the molecular profiling of tumours, including genomics, transcriptomics, proteomics, and metabolomics. This has led to the discovery of new biomarkers that may help detect prostate cancer earlier, predict its aggressiveness, and monitor the effectiveness of treatment. This review synthesizes current multi-omics data on exosomal cargo in prostate cancer, highlighting diagnostic, prognostic, and therapeutic implications as well as existing challenges to clinical translation.

## 1. Introduction

Prostate cancer (PCa) is one of the most prevalent malignancies affecting men worldwide and represents a major global health concern. It is estimated that there were 1.4 million new cases and 375,000 deaths from PCa worldwide in 2020 [1,2]. It ranks as the second most frequently diagnosed cancer in men worldwide, yet in Europe, it is the most commonly diagnosed malignancy and the third leading cause of cancer-related death among men [3], highlighting the urgent need for improved strategies in early detection, risk stratification, treatment and monitoring strategies. The incidence and mortality rates have been shown to be influenced by various factors, such as age, ethnicity, genetic background and staging [4].

Despite advances in screening and treatment, clinical challenges continue, primarily due to the heterogeneous nature of PCa and the limitations of existing diagnostic tools [5]. For decades, prostate-specific antigen (PSA) testing has formed the basis of screening and early detection of PCa. PSA is an enzyme in the glycoprotein family that is secreted by prostate epithelial cells, and small amounts are normally present in the bloodstream [6]. However, PSA testing is not cancer-specific: elevated PSA levels can also occur in conditions such as benign prostatic hyperplasia or prostatitis and can be caused by urological procedures [7]. This lack of specificity can result in false-positive findings, leading to unnecessary biopsies, patient anxiety, and the potential for overtreatment of indolent disease [8].

Over the past decade, extracellular vesicles (EVs) have emerged as a potential source of biomarkers for various diseases, among them PCa [9]. EVs represent a heterogeneous population of membrane-bound vesicles. Exosomes are a subtype of small EVs originating from endosomal multivesicular bodies and should be referred to as ‘small EVs’ when biogenesis cannot be confirmed.

Microenvironmental acidity has been shown to increase the secretion of PSA-positive and CD81-positive small extracellular vesicles, indicating that acidic tumor conditions promote vesicle release. This condition leads to the spill-over of nanovesicles into the blood, where the levels of tumour biomarkers expressed by these nanovesicles may represent a novel, non-invasive clinical tool for the screening and early diagnosis of PCa [10]. CA IX-positive exosomes were found to be much higher in PCa patients’ plasma samples than in those of healthy controls. Analysis of PCa-linked exosomes could help distinguish PCa patients from those with non-malignant prostatic disease, but exosome analysis is not yet standardised or cheap enough for large-scale use [11].

Defining an ‘abnormal’ PSA threshold is challenging due to the lack of universally standardised cut-off values [12]. PCa suspicion generally increases with serum PSA levels above age- and risk-adjusted thresholds (commonly 3–4 ng/mL). However, clinically significant cancers can occur even within these ranges, demonstrating the limitations of PSA-based screening [13]. When PCa is suspected based on elevated PSA levels or clinical findings, further evaluation typically involves multiparametric magnetic resonance imaging (mpMRI) to localise suspicious lesions, followed by a prostate biopsy for histopathological confirmation. They may also fail to capture tumour heterogeneity adequately. Setting a PI-RADS score of ≥4 as the threshold for biopsy indication significantly reduced the number of unnecessary biopsies and the detection of clinically insignificant PCa without compromising the detection of clinically significant PCa [14]. However, biopsies are invasive procedures that carry a risk of bleeding, infection, and urinary complications. MRI-based target biopsy can reduce the rate of adverse events by decreasing the number of cores obtained through omitting the systematic biopsy [15]. These diagnostic limitations highlight the need to develop new, minimally invasive biomarkers that enhance diagnostic accuracy, improve patient risk stratification, and inform personalized treatment strategies.

One promising approach is to integrate urine- and serum-based biomarkers alongside tissue-derived molecular markers to improve the early detection and classification of PCa. Using these advanced biomarkers could reduce unnecessary biopsies, more accurately identify clinically significant disease, and support the development of personalised management plans for patients [16].

Exosomes are nanoscale extracellular vesicles (30–200 nm) released into body fluids, where they transport molecular cargo reflective of their cell of origin. These organelles consist of different chemicals, including proteins, lipids, nucleic acids, and other substances such as amino acids and metabolites [17]. Exosomes play a key role in a variety of cellular processes and can transfer information between cells. Beit-Yannai et al. showed that several different cell types can secrete these exosomes which are enclosed by a single lipid bilayer membrane, which maintains cargo stability and facilitates intercellular transfer [18].

Exosomes play a role in regulating cancer growth by facilitating communication between cells. These cellular messengers transfer proteins and other biological substances through tissue fluids, thereby influencing the development of cancer [19]. Exosomes have the capacity to react to the growth and progression of tumor cells and can also influence the metastasis of tumor cells [20].

Recent progress in high-throughput sequencing, proteomic profiling, and exosome isolation technologies has fueled major interest in exosomes as components of liquid biopsy platforms. Instead of serving as liquid biopsies themselves, exosomes present in accessible fluids such as plasma and urine can be analyzed as part of minimally invasive liquid biopsy strategies.

These minimally invasive diagnostic approaches have the potential to complement, or in some cases even replace, traditional PSA testing and invasive tissue biopsies [21]. Exosome-based profiling has the potential to detect PCa at an earlier stage, predict how aggressive the disease is, help to decide on treatment, and monitor how the patient responds to treatment more accurately than conventional tools [22].

This review synthesizes current multi-omics data on exosomal cargo in PCa, highlighting diagnostic, prognostic, and therapeutic implications as well as existing challenges to clinical translation.

## 2. Exosomes

**Figure 1:**
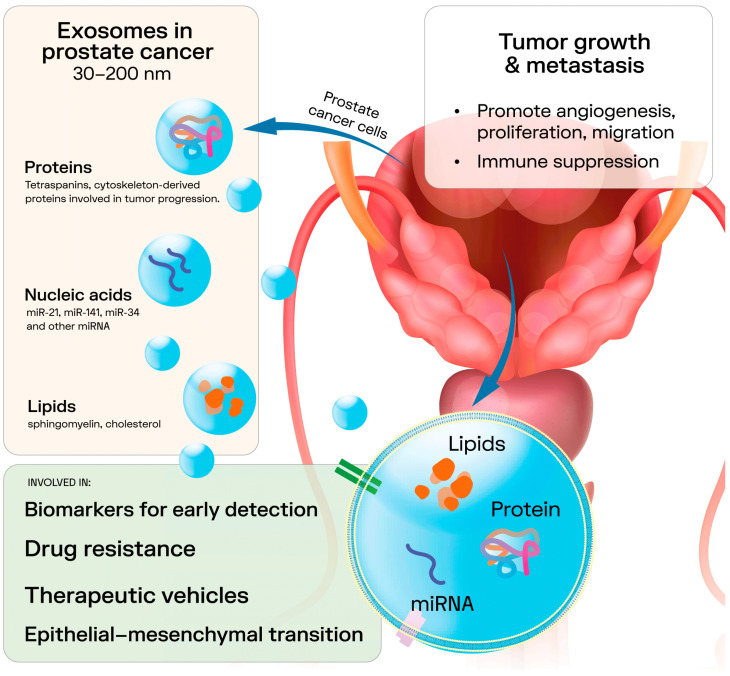
Role of exosomes in prostate cancer.

Exosomes are a prominent class of extracellular vesicles (EVs) measuring 30–200 nm. They are secreted by both eukaryotic and prokaryotic cells and are present in nearly all biological fluids [23,24]. They originate from the endosomal pathway, which allows for the incorporation of various biomolecules, including proteins (e.g., tetraspanin CD63, CD81, CD82, CD53 and CD37, as well as cytosolic proteins), lipids (e.g., sphingomyelin, cholesterol and generally saturated fats), nucleic acids, metabolites, and small molecules, either within their lumen or onto their membrane. This process facilitates intercellular communication [23]. Although the exact mechanisms of selective cargo loading are not fully understood, evidence suggests that the composition of exosomes varies depending on their cellular origin and the physiological or pathological state of the source cell [25]. This cargo variability reflects the status of the parent cells, highlighting exosomes as key players in disease modulation and promising biomarkers. Among their contents, membrane- and cytoskeleton-derived proteins [26] play a crucial role in cancer progression [27] and serve as diagnostic and prognostic markers [28]. Lipids, concentrated in the exosomal membrane, are vital for vesicle formation and stability in recipient cells, and their distinct profiles have also been linked to cancer regulation and diagnostics [28,29]. The size of exosomes influences the variation in their nucleic acids, including DNA and RNA. Larger exosomes tend to carry longer DNA fragments, while smaller vesicles are enriched in shorter nucleotide sequences [30]. EVs can deliver their cargo to recipient cells, thereby modulating signaling pathways [31]. For instance, exosomes from metastatic PCa cells are enriched with noncoding RNAs like miR-21 and miR-141, which regulate osteoclastogenesis and osteoblastogenesis. Tumor-derived exosomes also contribute to epithelial–mesenchymal transition (EMT) through miRNAs like miR-21 and miR-409, facilitating the progression from benign to malignant states [31]. Moreover, they influence drug resistance, as seen with miR-34 in PCa cells and exosomes targeting Bcl-2, which affect the response to docetaxel [32]. These findings underscore the pivotal role of EVs in PCa progression and therapy resistance (Figure 1).

## 3. Exosome Isolation and Identification

**Figure 2:**
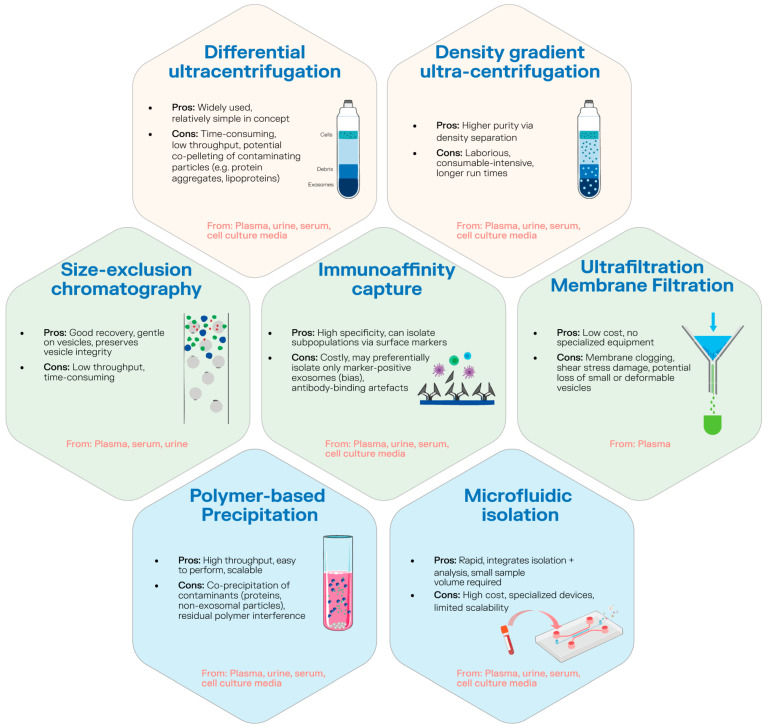
Methods for exosomes extraction.

Exosomes are found in all body fluids, including blood, urine, lymph, bile, saliva, milk, and amniotic fluid [17]. Their unique physicochemical features, including size, density, shape, charge, and surface antigens, allow for isolation through various methods such as size-exclusion chromatography, magnetic bead-based immunoaffinity, ultrafiltration, heparin affinity, and centrifugation techniques (Figure 2). Ultracentrifugation is the most commonly used method due to its simplicity, though it has both advantages and limitations. Despite significant advances in isolation strategies, achieving fully purified exosome preparations remains challenging [33].

After exosomes are isolated, various techniques are used to confirm their identity. These techniques are typically based on size, density, and specific markers [21]. Common approaches include dynamic light scattering (DLS) and nanoparticle tracking analysis (NTA) to determine size distribution [34], as well as electron microscopy methods, such as transmission electron microscopy (TEM), which is often combined with immunogold labeling [35], freezing electron microscopy, scanning electron microscopy (SEM), and atomic force microscopy (AFM) to characterize structure and morphology [36]. Biochemical assays such as Western blotting, the enzyme-linked immunosorbent assay (ELISA), and the photosensitizer magnetic bead detection system (ExoScreen) further assess purity and enrichment [37], while flow cytometry provides qualitative and quantitative insights, though it is limited by poor resolution for vesicles smaller than 100 nm [38]. Since each method has its own strengths and limitations, combining two or more techniques is often necessary for accurately characterizing exosomes.

A persistent barrier to clinical implementation is methodological heterogeneity in exosome research. Variability in isolation methods leads to inconsistent vesicle purity, yield, and molecular cargo composition, making it difficult to compare results across studies or establish reproducible biomarker signatures. Commonly used isolation techniques—including differential ultracentrifugation, density gradients, size-exclusion chromatography, polymer-based precipitation, and immunoaffinity capture—differ markedly in recovery efficiency and degree of contaminant co-isolation (e.g., lipoproteins, protein aggregates, apoptotic bodies). These differences directly influence downstream RNA, protein, and metabolite profiles, which in turn affect diagnostic and mechanistic interpretations. Compounding this challenge, pre-analytical factors such as sample collection time, anticoagulant choice, centrifugation speed, storage duration, and freeze–thaw cycles can introduce further variability in vesicle integrity and cargo stability. To advance toward clinical translation, the field must converge on standardized operating procedures for sample processing and vesicle isolation, incorporate certified reference materials to calibrate analytical assays, and adopt shared reporting frameworks such as those recommended by MISEV 2023. Harmonization of these methodological parameters, combined with multi-center validation and transparent experimental documentation, is essential to ensure the reproducibility, comparability, and ultimately, the clinical reliability of exosome-based biomarkers in PCa [39,40,41].

## 4. Tumor Microenvironment

The tumor microenvironment (TME) is a complex network consisting of tumor cells, infiltrating immune cells (e.g., macrophages, dendritic cells, and lymphocytes), cancer-associated fibroblasts, endothelial cells, lipids, the extracellular matrix, and signaling molecules [42]. The TME plays a critical role in drug resistance, immune suppression, and metastasis, making it a major focus of cancer research [43]. miR-203 in PCa cells’ exosome can change M0 macrophages to anti-tumour M1. This inhibits LNCAP cells proliferation, migration and invasion, whilst promoting apoptosis. In vivo studies show miR-203 as a therapeutic target. Its upregulation is associated with reduced tumour growth and increased M1 macrophages in the TME [44].

Within the TME, exosomal miRNAs act as key mediators of intercellular signaling that drive tumor progression, immune suppression, and metastatic niche remodeling. Beyond miR-21, miR-203 and miR-409 [31,44], several additional exosomal miRNAs have been implicated in PCa TME modulation. Exosomal miR-141 and miR-375 are enriched in metastatic and castration-resistant prostate cancer (CRPC) and promote osteoblast differentiation, facilitating osteotropic metastasis [45]. miR-92a and miR-27a, released from tumor cells and cancer-associated fibroblasts (CAFs), enhance angiogenesis through upregulation of VEGF and PI3K/AKT signaling in endothelial cells [46,47]. miR-146a and miR-125b act as immune regulatory miRNAs, suppressing T-cell–mediated cytotoxic responses and promoting M2 macrophage polarization. Additionally, exosomal miR-423-5p and miR-21 have been shown to induce fibroblast reprogramming into tumor-supportive CAFs, increasing extracellular matrix deposition and invasive potential [48,49]. Together, these exosomal miRNAs reprogram stromal, endothelial, and immune cells toward a tumor-permissive microenvironment and play a crucial role in metastatic dissemination, particularly to bone.

The tumor immune microenvironment (TIME) is closely related and strongly influences patient prognosis [50]. TIME consists mainly of bone marrow–derived cells and lymphocytes, and its activity depends on the types and functions of infiltrating immune cells, immune checkpoint expression, and matrix alterations [51]. Yang et al. investigated how PCa cells are attracted to osteoblasts by exosomes. Higher concentrations of CCL5 attract more PCa cells to the bone environment. Disrupting the circ-DHPS/miR-214-3p/CCL5 interaction may reduce cancer cell migration [52]. Dai et al. reported that high levels of miR183 enhance the invasion and migration of PCa cells by downregulating tropomyosin 1 (TPM1) [53]. miR-888 has been identified as a key player in promoting PCa cell growth and movement. PC3-ML cells contain high levels of exosomal miR-888, which downregulates proteins such as Krüppel-like factor 5 (KLF5), retinoblastoma-like protein 1 (RBL1), tissue Inhibitor of metalloproteinases 2 (TIMP2), and SMAD family member 4 (SMAD4), strengthening tumour cell abilities [54]. Osteoblasts play a pivotal role in PCa bone metastasis and are controlled by mechanisms found in exosomes. Studies have revealed a new mechanism involving exosomal miR-92a-1-5p in different PCa subtypes. It promotes osteoclast differentiation and suppresses osteogenesis. The bone-destructive potential of these subtypes correlates with levels of other osteogenic (miR-148a-3p, miR-375) and osteoclastic (miR-92a-1-5p) miRNAs [55]. Furthermore, PCa exosome-containing miR-26a-5p, miR-27a-3p, and miR-30e-5p suppress BMP-2-induced bone formation and osteoblast activity [56].

Both the TME and the TIME facilitate tumor progression, in part, through exosomes, which serve as key mediators of communication within these environments.

The role of exosomes in the tumor microenvironment directly informs their clinical potential as biomarkers for liquid biopsies. Because tumor-derived vesicles continuously circulate and mirror dynamic molecular changes occurring within the tumor niche, their cargo reflects ongoing processes such as immune evasion, epithelial–mesenchymal transition, and metabolic reprogramming. Therefore, mechanistic insights into exosome-mediated microenvironmental interactions serve as the biological foundation for their diagnostic and prognostic utility [21,57].

## 5. Exosomes’ Potential for PCa Diagnostics

**Figure 3:**
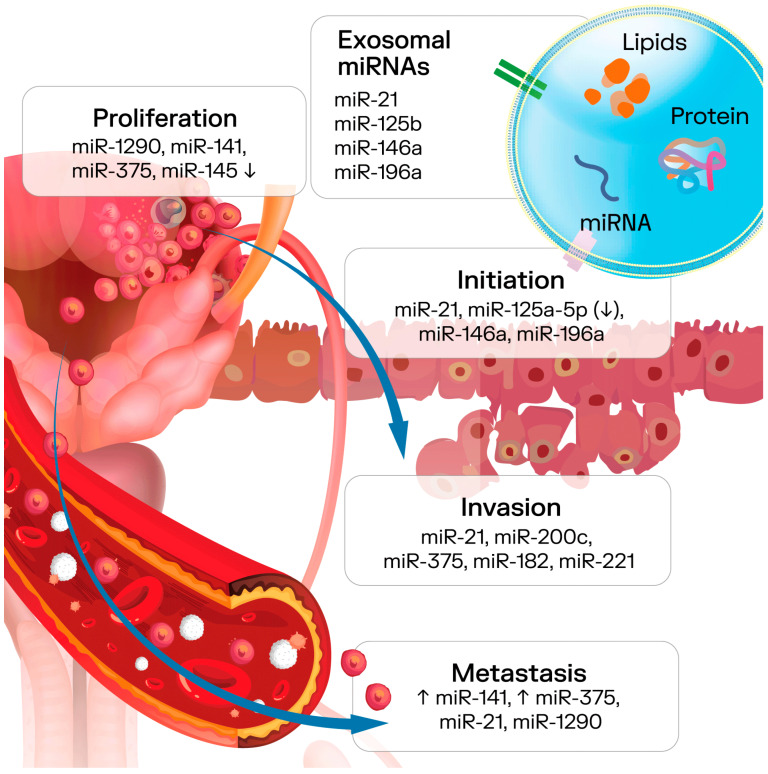
Role of miRNAs involved in exosomes cargo in different steps of tumor progression.

Most studies on exosomal biomarkers for PCa focused on miRNAs, with the majority reporting their overexpression, though some identified reduced levels of specific miRNAs in patients (Figure 3). In evaluations of diagnostic performance, exosomal miRNAs demonstrated superior diagnostic potential compared to the current gold standard, serum PSA analysis [58]. For example, high plasma exosomal expression of miR-141-3p and low expression of miR-125a-5p in PCa patients’ plasma exosomes might help identify specific tumour traits associated with PCa. Furthermore, the miR-125a-5p/miR-141-3p ratio appears to be a more effective marker than either value in isolation [59]. Exosomal miR-21, miR-141, miR-200c, and miR-375 have been consistently linked to disease progression across multiple studies and show great promise as non-invasive biomarkers for PCa, with potential applications in diagnosis, prognosis, therapy optimization, and prediction of clinical outcomes [60]. Urinary exosomal ERG, PCA3, PSMA, CK19, and EpCAM were significantly upregulated in PCa patients compared with healthy controls. Moreover, the expression levels of urinary exosomal ERG, ARV7, and PSMA showed a strong correlation with Gleason scores [61]. Urinary exosomal mRNA profiling, with an emphasis on RAB5B and WWP1, holds significant promise as a strategy to enhance the early detection of PCa [62].

Peng et al. identified four novel piRNAs with significant AUC values in ROC analysis [63], while Merkert et al. examined both piRNAs and miRNAs, reporting AUCs above 0.7 for most markers [64]. Other studies analyzed lncRNAs in blood and urine samples (SAP30L-AS1, SChLAP1, AC015987.1, CTD-2589M5.4, RP11-363E6.3), all showing significant overexpression in PCa [65,66]. Urinary exosomal lncRNA assay combining PCA3 and MALAT1, which outperformed current clinical parameters by achieving a higher AUC for predicting biopsy outcomes and detecting high-grade disease [67]. Protein expression was assessed using Western blot, ELISA, or SDS-PAGE, focusing mainly on cancer-related proteins (PSMA, PCA3, TMPRSS2:EGR, PSA) or exosomal surface proteins (CD9, CD3, EpCAM, CD81) [68]. Cytokines were identified as promising diagnostic markers [69,70], and elevated levels of enzymes such as carbonic anhydrase IX and gamma-glutamyltransferase were also reported in PCa patients [71,72]. Clinical studies have also examined EV-derived RNAs as biomarkers: Yang et al., through meta-analysis, confirmed the strong diagnostic value of plasma exosomal miRNAs in PCa [73]. The early diagnosis of PCa can be facilitated by the combined prediction models, which consist of plasma exosomal miRNAs (hsa-miR-320c and hsa-miR-944), US radiomics, and clinical tPSA [74]. The use of advanced tools, including AI- and nanotechnology-based sensors, improves the accuracy of PCa diagnostics centred on exosomes, resulting in more sensitive detection and superior clinical outcomes [75].

A growing number of studies have investigated exosomal biomarkers in different biofluids, particularly plasma, urine, and semen, reflecting the accessibility and clinical relevance of these sample types in PCa, as summarized in Table 1. Plasma-derived exosomal miRNAs such as miR-141 and miR-375 have been repeatedly associated with metastatic and castration-resistant disease, while urinary exosomal lncRNAs such as PCA3 and PCAT-1, and protein cargo such as PSMA, have demonstrated utility in distinguishing PCa from benign prostatic hyperplasia. Exosomal miRNAs, lncRNAs, proteins, and metabolites and their clinical implications in PCa are represented in Table 2. Seminal fluid exosomes are especially enriched in prostate-derived vesicles and therefore provide a more tissue-specific signal; however, systematic clinical evaluation remains limited. Importantly, while these biomarkers show promising diagnostic and prognostic performance within individual studies, direct comparisons across studies reveal substantial variability in sensitivity, specificity, and threshold values. These discrepancies are primarily driven by inconsistencies in pre-analytical handling (e.g., centrifugation steps, storage time, and temperature), isolation method (ultracentrifugation vs. size-exclusion chromatography vs. polymer-based precipitation), and downstream quantification platforms (qPCR vs. NGS vs. digital PCR). Moreover, the absence of universally accepted endogenous reference controls for exosomal RNA normalization significantly limits reproducibility and cross-cohort comparability. Notably, biomarker panels that perform well in discovery cohorts often lose predictive power in external validation settings, underscoring the need for multi-center standardization. Therefore, while exosomal cargo holds strong potential for clinical integration as a non-invasive diagnostic platform, rigorous standardization of workflows, transparent reporting of analytical parameters, and validation in large, prospectively collected cohorts are essential to establish clinical reliability and regulatory feasibility.

In the coming years, collaborative efforts among clinicians, researchers, and bioinformaticians will be crucial in determining which patients should undergo molecular profiling and when, designing more effective, biomarker-driven clinical trials, addressing technical challenges, and ultimately integrating liquid biopsy into the routine clinical care of PCa patients [85,86].

## 6. Exosomes’ Potential as a PCa Therapy

Two glycoproteins, P-glycoprotein and oncofetal glycoprotein 5T4, were among the quantified proteins and were linked to docetaxel resistance and the persistence of malignant cells, respectively [87]. Vardaki et al. reported that the immune checkpoint protein PD-L1 was associated with shorter overall survival in patients treated with Radium-223 [83]. Exosomal microRNAs play a role in PCa chemoresistance [88] and may serve as surrogate biomarkers for tumor response to taxane-based therapies [89]. Studies have shown that exosomes—particularly those carrying MDR-1/P-gp—from docetaxel-resistant cell lines can transfer resistance to sensitive PCa cell lines such as DU145, 22Rv1, and LNCap [90]. Similarly, Kawakami et al. identified β4 (ITGB4) and vinculin within exosomes as potential markers of PCa progression, closely linked to taxane resistance [91]. Notably, elevated serum exosomal P-glycoprotein levels correlate with resistance to docetaxel but not to cabazitaxel, suggesting its value as a biomarker to guide treatment decisions in PCa management [87]. CAF-derived exosomal miR-196b-5p, upregulated after androgen deprivation therapy, promotes epithelial–mesenchymal transition in PCa cells via the HOXC8/NF-κB pathway, revealing a mechanism of metastasis and potential therapeutic targets for post-castration management [92]. Liu et al. reported that PCa cell–derived exosomes upregulated PD-1 and TIM-3 expression in CD8+ T cells, promoted the release of cytokines associated with T cell exhaustion, and markedly reduced their cytotoxic activity against PCa cells. Treatment with GW4869 reversed these effects by inhibiting exosome production, thereby restoring CD8+ T cell function and suppressing PCa cell growth both in vivo and in vitro. These results suggest that GW4869 may hold therapeutic potential for PCa [93]. DU145 cells are more radioresistant than PC3 cells, and exosomes may contribute to this resistance [94]. Therefore, investigating exosome functions is key to understanding carcinogenesis and improving radiotherapy. One study showed that urinary miR-664a-5p was significantly upregulated in patients responding to PARP inhibitors [95]. In addition, Pukha et al. identified specific metabolites (glucuronate, D-ribose 5-phosphate, and isobutyryl-L-carnitine) as potential markers of successful prostatectomy [96], while Macías et al. demonstrated that lncRNAs (CCL2, CXCL5, and S100A9) could predict surgical efficacy and recovery [70]. Androgen receptor splice variant 7 (AR-V7) expression resulted in a strong predictor of response to ARSIs and hormone therapy [97]. Malla and colleagues reported that miRNAs let-7a-5p and miR-21-5p were elevated in high-risk PCa patients undergoing radiotherapy compared to those with intermediate-risk disease [98]. Considering the limited effectiveness of current immunotherapies for PCa, targeting PD-1–carrying exosome secretion or inhibiting USP7 function could represent promising immunostimulatory strategies for treatment [99].

## 7. Exosomes’ Potential as a PCa Prognostic

Exosomal cargo detected through liquid biopsy has strong prognostic potential, as it can indicate disease grade, metastatic risk, overall survival, and biochemical recurrence-free survival. Among these, specific miRNAs and lncRNAs stand out, showing significant associations with tumor aggressiveness and recurrence. Gao et al. similarly highlighted the value of exosomal miRNAs in tracking PCa invasion and metastasis [100]. Using exosomal biomarkers to stratify patients by Gleason score and predict biochemical recurrence could transform active surveillance, enabling more personalized PCa management [101]. In a large cohort, Wang et al. used the Sentinel™ platform to identify three sncRNAs with superior sensitivity and specificity for diagnosing and predicting high-grade PCa [102]. Additionally, circRNAs were evaluated, all of which showed significant associations with prognosis [103]. Zavridou et al. linked GSTP1 and RASSF1A gene methylation with overall survival [104], while Tao et al. identified several lncRNAs (AC015987.1, CTD-2589M5.4, and RP11-363E6.3) as potential tools for guiding active surveillance [66]. Similarly, Kretschmer et al., using the ExoDx test in over 2000 patients, demonstrated its value in stratifying disease from grade group 1 to 3 and supporting surveillance strategies [105]. Overall, most studies reported that elevated exosomal biomarker expression correlates with poor outcomes or higher-grade disease, except Ruiz-Plazas et al., who observed reduced miRNA expression linked to high-risk cases [106]. Wang et al. reported that exosome-associated genes, including AQP2 and ZNF114, show strong potential as non-invasive biomarkers for predicting PCa status and prognosis, offering an alternative to highly invasive diagnostic procedures [107]. Urinary exosomal FAM153C-RPL19 demonstrated greater diagnostic value than PSA, particularly in gray-zone cases, with elevated levels linked to poor prognosis [108].

While exosome-derived biomarkers show promising diagnostic and prognostic potential in PCa, translation into routine clinical use remains limited by several methodological constraints. A key challenge is the lack of standardization across pre-analytical workflows, including biological fluid selection, time-to-processing, and freeze–thaw conditions, all of which influence vesicle integrity and detected cargo profiles. Divergent exosome isolation methods (ultracentrifugation, size-exclusion chromatography, polymer-based precipitation, and immunoaffinity capture) yield heterogeneous vesicle populations with variable purity. Moreover, many studies rely on limited patient cohorts without external validation, reducing reproducibility and generalizability. Multi-center validation studies paired with MISEV 2023-aligned reporting standards are necessary to establish clinically robust biomarker signatures [109].

## 8. Conclusions and Perspectives

Although exosomal biomarkers hold significant promise for improving prostate cancer detection, risk stratification, and treatment monitoring, their translation into routine clinical practice remains challenging. A critical barrier is the lack of standardized isolation and characterization workflows, which results in substantial variability in vesicle purity, yield, and molecular cargo profiles across laboratories. This methodological heterogeneity complicates biomarker comparison, hinders reproducibility, and weakens the strength of evidence required for regulatory approval. Furthermore, many studies have been conducted in small or demographically narrow patient cohorts, often without external or prospective validation, limiting generalizability across disease stages and populations. Comprehensive multi-center studies using harmonized analytical pipelines and clinically annotated biospecimen collections will be essential to define clinically robust exosomal signatures. In addition, integration of exosome-based biomarkers with existing clinical frameworks—including risk calculators, imaging modalities, and molecular classifiers—will be necessary to determine their incremental predictive value over current standards of care. Advances in digital PCR, single-vesicle analysis, and machine learning–based biomarker modeling may further enhance precision and interpretability. Ultimately, the successful clinical adoption of exosome-based diagnostics will require a convergence of methodological standardization, large-scale validation, and incorporation into evidence-based clinical decision-support systems.

## Figures and Tables

**Table 1:** Potential miRNA biomarkers for prostate cancer in clinical settings.

Year	Exosomal Biomarkers	Sample Source	Exosomal Contents	Clinical Practice	Change in Expression	Reference
2017	miR-196a-5p, miR-34a-5p, miR-143-3p, miR-501-3p, miR-92a-1-5p	Urine	miRNAs	20 PCa vs. 9 healthy people	Reduced	[76]
2017	miR-21, miR-141, miR-375	Urine	miRNAs	60 PCa vs. 10 healthy people	Increased	[77]
2020	(miR-125a-5p/miR-141-3p) ratio	Plasma	miRNAs	31 PCa vs. 19 healthy people		[59]
2022	lncRNA-p21	Urine	lncRNAs	Identify PCa with benign disease	Increased	[78]
2017	miR-375	Serum	miRNAs	50 PCa vs. 22 BPH	Increased	[79]
2019	(PSA + miR-142-3p + miR-142-5p + miR-223-3p) panel	Semen	miRNAs	31 PCa vs. 24 BPH	Increased	[80]
2019	Survivin	Plasma	miRNAs	Early detection of PCa	Increased	[81]

**Table 2:** Exosomal miRNAs, lncRNAs, proteins, and metabolites to clinical implications in PCa.

Cargo Type	Representative Molecules	Molecular/Functional Role	Disease Context in Prostate Cancer	Clinical Implication	Key References
miRNAs	miR-141, miR-21, miR-375, miR-1290, miR-423-5p	Regulate EMT, AR signaling, cell survival, immune modulation	Elevated in metastatic and CRPC patients; associated with treatment resistance and tumor aggressiveness	Diagnostic: Distinguish PCa vs. benign disease; Prognostic: Predict metastatic progression and biochemical recurrence	[48,77,82]
lncRNAs	PCA3, SChLAP1, MALAT1, PCAT-1	Compete with miRNAs, alter chromatin remodeling, modulate AR co-regulators	Overexpressed in high-risk PCa and detectable in urinary exosomes	Non-invasive diagnosis (especially urine-based tests), risk stratification	[65,66,67]
Proteins	PSA, PSMA, PD-L1, ITGB4, CD44v6	Promote immune evasion, metastatic adhesion, chemoresistance signaling	Elevated in exosomes from drug-resistant tumor cells; PD-L1+ exosomes correlate with immune suppression	Predictive biomarker for therapy response (ADT/AR-targeted therapy/docetaxel); potential therapeutic targets	[68,83,84]
Metabolites & Enzymes	Carbonic anhydrase IX, γ-glutamyltransferase (GGT), Lactate-associated metabolites	Regulate extracellular acidification, oxidative stress adaptation, metabolic reprogramming	Support tumor cell survival under hypoxia, promote bone niche formation in metastasis	Prognostic markers for aggressiveness; potential targets for metabolic intervention	[71,72]

## Data Availability

No new data were created or analyzed in this study.
